# Hypertension and Dementia in the Elderly: The Leisure World Cohort Study

**DOI:** 10.1155/2012/205350

**Published:** 2011-12-20

**Authors:** Annlia Paganini-Hill

**Affiliations:** ^1^Department of Preventive Medicine, Keck School of Medicine, University of Southern California, Los Angeles, CA 90033, USA; ^2^Department of Neurology University of California, Irvine CA 92697, USA

## Abstract

Recent studies have highlighted the deleterious role of cardiovascular risk factors, including hypertension, on the incidence of dementia. Although midlife hypertension is associated with later development of dementia, the role of late-life hypertension remains unclear. We explored the association of hypertension and its treatment with incident dementia in 13978 older (median = 74 years) adults followed from 1981 to 2010 (median = 13 years) and calculated risk estimates using Cox regression analysis in two age groups (<75 and 75+ years) in men and women separately. Dementia status was determined from in-person evaluations, followup questionnaires, hospital data, and death certificates. In the older women, current users of blood pressure medication at baseline had a 26% increased risk of dementia (95% CI 1.06–1.51). In the younger men, those with untreated hypertension and those with past use of blood pressure medication use had about a 30% nonsignificant increased risk of dementia. High blood pressure and its treatment appear to have different effects in men and women and in the old and older.

## 1. Introduction

As a result of an aging population, the prevalence of dementia, which in 2005 affected 24.3 million people worldwide, is expected to afflict more than 81 million by 2040 [1]. Recent studies have highlighted the deleterious role of cardiovascular risk factors, including hypertension, on the incidence of dementia and suggested that their therapeutic control may reduce risk of development of dementia in later life [2, 3]. Several longitudinal studies have found midlife hypertension to be related to dementia (reviewed in [3, 4]), but the role of late-life hypertension remains unclear. 

The focus of the present study was to examine the possible role of hypertension and its treatment as predictors of dementia in elderly men and women. We report here the results in a large cohort (nearly 14000) of elderly (median age 74 years) men and women followed for up to 29 years (median 13 years).

## 2. Methods

The Leisure World Cohort Study was established in the early 1980s when 13978 residents of a California retirement community (Leisure World Laguna Hills) completed a postal health survey. Residents were recruited in four waves: those who owned homes in Leisure World on June 1, 1981; new residents who had moved into the community and were living there on June 1, 1982; on June 1, 1983; on October 1, 1985. The baseline survey asked demographic information, brief medical history, medication use, and personal habits including cigarette smoking, exercise, alcohol consumption, and beverage intake. The subjects were asked if a doctor ever told them they had high blood pressure and if they had ever taken or were currently taking specific medications including “Reserpine (please include Raudixin, Ser-Ap-Es, Hydropres, Rauwolfias, Metatensin)” and “other blood pressure medication, water pills.” The population and the cohort are mostly Caucasian, well educated, upper-middle class, and elderly.

 Followup of the cohort is maintained by periodic resurvey (1983, 1985, 1992, 1998), review of local hospital discharge data (1981–2001), and determination of vital status by search of governmental and commercial death indexes and ascertainment of death certificates. The 1983 followup questionnaire asked new diagnoses of high blood pressure since the baseline survey. The 1998 questionnaire again asked if the subject had ever been told by a doctor that they had high blood pressure and the frequency of use of high blood pressure medication.

Dementia cases were identified from in-person evaluations as part of a dementia study [5], hospital records, death certificates, and/or followup questionnaires with the date of diagnosis being the date at which dementia was first mentioned. Participants were followed to dementia diagnosis, death or December 31, 2010, whichever came first. To date 37 cohort members have been lost to followup; search of death indices did not reveal that these individuals were deceased.

Chi-square tests were used for comparison of categorical variables and *t*-tests for testing differences in means of continuous variables. Age-adjusted hazard ratios (HRs) of dementia associated with hypertension were estimated using Cox proportional hazard regression analysis with age at study entry being the age when the baseline survey was completed and the event of interest being age at dementia. Separate analyses were done for two age groups: <75 years and 75+ years of age at baseline, with additional adjustment for age (continuous). Because women differ from men on many variables, women live longer on average than men, and for comparison with other studies limited to one sex, we performed separate analyses for men and women. Statistical analyses were performed using SAS version 9.2 (SAS Institute Inc., Cary, NC). No adjustment in the *P* values was made for multiple comparisons.

Previous reports present details of the methods and the reliability of recall of the self-reported information [6–9]. The Institutional Review Boards of the University of Southern California and the University of California, Irvine approved the study.

## 3. Results

At study entry, the participants' mean and median age was 74 years (standard  deviation = 7). By December 31, 2010, 2429 participants had been diagnosed with dementia and 13134 had died. Age at dementia ranged from 66 to 106 years (median, 90 years). Dementia cases were identified by The 90+ Study (*n* = 690), hospitalization record (*n* = 199), followup questionnaire (*n* = 264), and death certification (*n* = 1768); these categories are not mutually exclusive.

Characteristics of the study participants are shown in Table 1. Women differed significantly from men on all these variables (*P* < 0.001 for all variables except cancer where *P* < 0.02).

Tables 2 and 3 show the age-adjusted HRs of incident dementia for hypertension and its treatment for women and men, respectively. Forty-three percent of subjects reported not having high blood pressure and not taking hypertensive medication. Of the remaining with high blood pressure, about 21% took no medication, 18% took medication in the past but not currently, and 18% were currently taking blood pressure medication. The only significant risk factor for dementia was current blood pressure medication (HR = 1.26, 95% CI 1.06–1.51) in women aged 75+ years. No effect of current treatment was seen in men. The greatest (though nonsignificant) risks in men were found among men less than 75 years old with untreated hypertension (HR = 1.27, 95% CI 0.95–1.70) and with past use of blood pressure medication (HR = 1.33, 95% CI 1.00–1.79). 

Followup questionnaires identified few additional individuals with hypertension. Of the 9731 who returned the 1983 followup survey, 188 (2%) reported a new diagnosis of high blood pressure. In 1998, 219 (11%) of 1963 who returned the questionnaire reported having high blood pressure in 1998 but not at baseline. 

## 4. Discussion

In recent years the strict division between Alzheimer's disease and vascular dementia has faded with advancing research in neuropathology, neuroradiology, and epidemiology. Most dementia patients, irrespective of their clinical diagnosis, have mixed pathology (Alzheimer's changes and cerebrovascular lesions) at autopsy [10, 11]. High blood pressure has long been understood to cause stroke, a risk factor for vascular dementia. However, the association between blood pressure and dementia is complex. Midlife hypertension increases risk of cognitive impairment, Alzheimer's disease, and dementia (reviewed in [3, 4]). 

Associations in longitudinal studies of late-life hypertension and dementia have been less consistent. Some found blood pressure levels were higher in individuals who developed dementia [12], Alzheimer's disease [13], or vascular dementia [14] than in those who did not. In the Swedish Longitudinal Population Study of 382 participants (aged 70 years) subjects who developed dementia had significantly higher systolic and diastolic blood pressures about 10 to 15 years before cognitive assessment than subjects without dementia [12]. In the Kungsholmen Project, a community-based cohort of 1270 participants (aged ≥75 years) in Sweden followed for 6 years, 339 subjects were diagnosed with dementia [13]. Subjects with high SBP (>180 mmHg) had a risk of 1.6 (95% CI 1.1–2.2) for dementia. High DBP (>90 mmHg) was not associated with increased risk. In Hisayama Study, Japan, in which 828 people (aged 65–98 years) were followed for 7 years, blood pressure was not related to AD but 1 standard deviation increase in SBP increased risk of vascular dementia 60% (95% CI 1.2–2.2) [14]. The Adult Changes in Thought Study included 2356 participants aged ≥65 years followed for 8 years during which time 380 developed dementia [15]. Only in the youngest age group (65–74 years) did those with high SBP (≥160 mmHg) have a significantly increased risk of dementia (HR 1.6, 95% CI 1.01–2.55) and the risk declined with advancing age to 0.64 (95% CI 0.32–1.30) in the oldest age group (>85 years). In the Women's Memory Study blood pressure levels measured 5 and 9 years before dementia assessment did not differ significantly between demented and nondemented [16]. Others have observed that history of hypertension was not related to dementia [17, 18], Alzheimer's disease [19–22] but was related to vascular dementia [22]. Interestingly, in the Canadian Study of Health and Aging, the presence of hypertension did not result in cognitive deterioration across the cohort of subjects (mean age 83 years) [23]. However, subjects with hypertension and cognitive executive dysfunction increasingly progressed to dementia (58% in five years) compared with normotensives (28%) but not in those with hypertension and memory dysfunction (74% versus 67%). 

Our study extends the available literature on the role of hypertension and dementia in the very elderly but has several limitations. Information on hypertension and blood pressure medication was self-reported; we performed no blood pressure determinations. However, previous studies in our population and others support the reliability of self-reported health practices, drug usage, and medical history of major chronic disease [8, 9]. Although changes over time in all potential risk factors may affect outcome, new cases of hypertension were reported in only a small minority of subjects. Additionally, the proportion of demented individuals is undoubtedly an underestimate of the true incidence, and we lacked clinical diagnoses of dementia based on standard criteria for many subjects. Subjects with dementia who had not been seen in person or hospitalized and were not reported as having dementia at the time of the followup surveys or on their death certificates were actually misclassified into the nondemented group. This has, with all likelihood, weakened the associations seen here. For these reasons our results are preliminary and incomplete. Furthermore, our subjects are from a select population—moderately affluent, highly educated, health conscious, and primarily Caucasian. Although this may limit the generalizability of our results, it reduces potential confounding by race, education, SES, and presumed access to health care. Nonetheless, unrecognized and uncontrolled confounders cannot be ruled out in this study or any observational study. 

Clinical trials of antihypertensive medication in older subjects have also yielded inconsistent results (reviewed by [2–4]). Randomized controlled trials have demonstrated that blood pressure-reducing agents decrease the incidence of dementia in stroke patients (PROGRESS [24], HOPE [25]) and in older (≥60 years) patients with isolated systolic hypertension (SYST-EUR) [26, 27], but this was not found in SHEP [28], SCOPE [29], and HYVET-COG [30]. These latter three included more elderly patients. The SHEP (Systolic Hypertension in the Elderly Programme) trial of 4736 patients (mean age 72 years) found a nonsignificant 16% reduction in dementia in treatment groups. The SCOPE (Study on Cognition and Prognosis in the Elderly) study of 4964 elderly (mean age 76 years) and HYVET-COG (Hypertension in the Very Elderly Trial-cognitive function assessment) of 3336 patients aged ≥80 years found no effect on cognitive decline, Alzheimer's disease, or vascular dementia. Since different antihypertensive medications were used by our study subjects as well as in the clinical trials which looked for an effect of medication on dementia development, a specific class effect cannot be ruled out.

Others have detailed the possible mechanisms by which hypertension may increase risk of dementia (reviewed in [3, 4]). Hypertension may promote blood vessel wall thickening leading to arteriosclerosis and lipohyalinosis; cause stroke, focal ischemia, chronic hypoperfusion of the white matter, and white matter lesions; cause microcirculation disorders and endothelial dysfunction which may compromise the function of the blood brain barrier leading to increased vascular permeability and extravasation of protein into the cerebral parenchyma.

Although not establishing a cause and effect relationship between incident dementia and high blood pressure and its treatment, this large elderly cohort suggests an increased risk of dementia in the younger (<75 years) men with untreated hypertension and with past use of hypertensive medication. This agrees with the findings of earlier studies showing an increased risk of dementia with higher measured blood pressure levels. In women the risk among current (at baseline) users of blood pressure medication, which was the strongest in the older age group (75+ years), highlights the need for careful monitoring of blood pressure in the elderly. Decline in blood pressure is common at ages above 75 years, and the Kungsholmen Project found low blood pressure to be associated with dementia risk (HR = 1.5, 95% CI 1.0–2.1) [13]. Periods of hypotension, hypoperfusion and hypoxia observed in subjects on antihypertensives might contribute to cognitive decline via reduced cerebral blood flow, causing ischemic lesions in the brain. 

## 5. Conclusion

Although treating hypertension has a clear effect on stroke, cardiovascular disease, and mortality, the effect of hypertension and its treatment on dementia is more complex. While previous studies have found midlife hypertension to be related to dementia, results for late-life hypertension and its treatment are inconsistent. Our study in older adults suggests that the effect varies not only with age (with a difference between those less than 75 years old and those 75+ years old) but also with sex. Future studies examining the role of hypertension in older adults should perform sex-specific analyses.

## Figures and Tables

**Table 1:** Characteristics of participants by sex, The Leisure World Cohort.

Number	Men		Women	
	5101		8877	
	Mean	SD	Mean	SD
Age at baseline (years)	74	7.2	73	7.4
Age at last followup (years)	86	7.0	88	7.0
Followup years	11	7.3	15	7.7

Active activities (hrs/day)	1.1	1.3	0.9	1.1
Other less strenuous activities (hrs/day)	3.6	2.7	4.4	2.6
Alcohol (drinks/day)	1.6	1.5	1.2	1.2
Caffeine (mg/day)	176	172	168	166
Body mass index (kg/m^2^)	24	2.9	23	3.5

	No.	%	No.	%

Medical history				
High blood pressure	1843	36	3620	41
Angina	738	14	839	9
Heart attack	847	17	588	7
Stroke	370	7	333	4
Cancer	826	16	1574	18
Diabetes	425	8	442	5
Rheumatoid arthritis	226	4	608	7

Smoke				
Never	1708	33	4883	55
Past	2953	58	2880	32
Current	437	9	1106	12

Deceased by December 31, 2010	4930	97	8204	92

**Table 2:** Hazard ratios for dementia by hypertension and its treatment in men, Leisure World Cohort Study, 1981–2010.

	Aged <75 years				75+ years			
	No.	No. with dementia	HR	95% CI	No.	No. with dementia	HR	95% CI
Hypertension and treatment								
No hypertension and no treatment	1160	153	1.00		1171	163	1.00	
Hypertension but no treatment	495	66	1.27	0.95–1.70	480	51	1.02	0.75–1.41
Past treatment	449	64	1.33	1.00–1.79	384	42	0.92	0.66–1.30
Current treatment	430	42	1.00	0.71–1.41	532	56	1.01	0.74–1.37

Current hypertensive medication								
No	2104	283	1.00		2035	256	1.00	
Yes	430	42	0.89	0.64–1.24	532	56	1.02	0.76–1.36

**Table 3:** Hazard ratios for dementia by hypertension and its treatment in women, Leisure World Cohort Study, 1981–2010.

	Aged <75 years				75+ years			
	No.	No. with dementia	HR	95% CI	No.	No. with dementia	HR	95% CI
Hypertension and treatment								
No hypertension and no treatment	2131	479	1.00		1522	345	1.00	
Hypertension but no treatment	1054	177	0.88	0.74–1.05	937	155	0.91	0.76–1.11
Past treatment	1011	202	0.90	0.77–1.06	685	137	0.92	0.75–1.12
Current treatment	750	148	0.99	0.82–1.19	787	149	1.21	1.00–1.47

Current hypertensive medication								
No	4196	858	1.00		3144	637	1.00	
Yes	750	148	1.04	0.87–1.24	787	149	1.26	1.06–1.51
